# Multiple Roles of Cannabinoids in the Olfactory System

**DOI:** 10.3390/brainsci16020190

**Published:** 2026-02-05

**Authors:** Thomas Heinbockel, Edward A. Brown

**Affiliations:** Department of Anatomy, Howard University College of Medicine, Washington, DC 20059, USA

**Keywords:** cannabinoid, endocannabinoid system, olfactory system, olfaction, CB1R, CB2R, retrograde signaling

## Abstract

The endocannabinoid system is a ubiquitous neuromodulatory network that links internal physiological state to neural circuit function across the brain. While its roles in memory, reward, pain, and motor control are well established, its contribution to olfactory processing has only recently gained attention. This review synthesizes the current knowledge on the anatomical, cellular, and functional interactions between the endocannabinoid system and the olfactory pathway, from the olfactory epithelium and main olfactory bulb to higher order cortical targets. We highlight how endocannabinoid signaling, primarily via cannabinoid receptor type 1 (CB1), shapes synaptic transmission within olfactory bulb microcircuits, modulates centrifugal feedback, and adjusts sensory gain in a state-dependent manner, particularly in relation to hunger, feeding behavior, stress, and reward. In addition, we review evidence that the endocannabinoid system regulates olfactory neurodevelopment and adult neurogenesis by influencing neural stem cell proliferation, migration, and integration into existing circuits. Emerging links between endocannabinoid signaling, olfactory dysfunction, neuropsychiatric disease, metabolic disorders, and neurodegeneration underscore the translational relevance of this system. We also discuss methodological challenges inherent to studying endocannabinoid signaling and outline future directions, including circuit-specific targeting and intranasal delivery strategies. Together, these findings position the olfactory system as a powerful and accessible model for understanding how endocannabinoids couple internal state to perception and behavior, with important implications for therapeutic development.

## 1. Introduction

Brain diseases such as epilepsy, stroke, pain and mental brain disorders such as schizophrenia continue to have a major impact on brain health. In the 1990s, endocannabinoids, the endogenous correlate of the neurochemical compound found in the plant *Cannabis sativa*, were isolated and found to play a neurochemical role throughout the brain and body [1,2]. At this point in history, there were public debates about whether the drug marijuana (cannabis) should be made legal. Parts of the public were advocating for cannabis legalization and decriminalization, which could lead to changes in its classification as a controlled substance. While cannabis is federally illegal, many states in the United States have legalized or decriminalized it for recreational or medical use. For some people, cannabis shows medicinal benefits while for others it serves recreational purposes. Research has pointed to potential health benefits, especially brain health benefits of the endocannabinoid system and cannabis sativa. In the United States, 24 states, two territories, and the District of Columbia have legalized recreational cannabis while medical cannabis is legal in 40 out of 50 states [3]. The endocannabinoid system is a neuromodulatory system that regulates internal physiological states and neural circuit function. The contribution to olfactory processing has only recently gained attention. Endocannabinoid signaling, primarily via cannabinoid receptor type 1 (CB1), shapes synaptic transmission within olfactory bulb microcircuits, modulates centrifugal feedback, and adjusts sensory gain in a state-dependent manner, particularly in relation to hunger, feeding behavior, stress, and reward. The endocannabinoid system also regulates olfactory neurodevelopment and adult neurogenesis by influencing neural stem cell proliferation, migration, and integration into existing circuits.

Here, we discuss the recent progress in understanding the role of the endocannabinoid system in the olfactory pathway, the interactions between the two systems, the potential implications for the relevance of the endocannabinoid system in clinical settings, methodological limitations, and outline future research directions.

## 2. Endocannabinoid System Biology

THC, tetrahydrocannabinol, the exogenous cannabinoid isolated from the plant *Cannabis sativa* [2,4,5], was found in the late 1990s to have endogenous correlates in the form of two compounds that have come to be known as endocannabinoids because they are produced endogenously by the human body. The two different endocannabinoids are *N*-arachidonoylethanolamide, (anandamide, AEA) and 2-arachidonoylglycerol (2-AG). Two endocannabinoid receptors have been described: cannabinoid 1 receptor, CB1R [6], which is found primarily in the brain, and cannabinoid 2 receptor, CB2R, found primarily in the immune system. Unlike neurotransmitters synthesized in the cell soma and transported down to the axon terminal and stored there until synaptic release is triggered, endocannabinoids are formed upon demand from the membrane lipid bilayer [7]. A second unique quality of endocannabinoids is that rather than being released from a presynaptic cell onto a postsynaptic cell, endocannabinoids are released from the postsynaptic cell and then bind onto receptors on the presynaptic cell. This function of the cellular signaling system, called retrograde signaling, is to allow the modification of signal input onto the postsynaptic cell by the presynaptic cell [7,8,9,10]. Examples of this type of retrograde signaling have been found in several brain regions and termed Depolarization-induced Suppression of Inhibition (DSI). Postsynaptic endocannabinoid release results in the inhibition of a presynaptic inhibitory cell. DSI was identified in GABAergic neurons of the hippocampus, cerebellum, amygdala, and dopaminergic neurons of the ventral tegmental area [7,11,12]. A second signaling system is termed Depolarization-induced Suppression of Excitation (DSE), in which postsynaptic endocannabinoid release results in the inhibition of an excitatory cell as found in the cerebellum [13,14].

Key functions of the endocannabinoid system have been observed in brain regions that include the hippocampus, amygdala, and cerebellum. Until recently, an open question in cannabinoid research was the relevance of the endocannabinoid system for olfactory processing. This review aims to provide answers to this question: What unique interactions can be found between the cannabinoid and the olfactory system [15,16,17,18,19,20]? Through a greater understanding of the role of the endocannabinoid system, new brain treatments and therapies [21,22], as well as more responsible usage of the exogenous form, *Cannabis sativa*, might be achievable.

**Figure 1:**
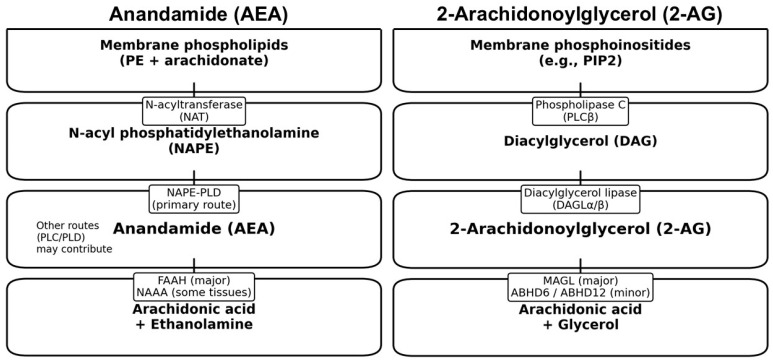
Core synthesis and degradation pathways for the two major endocannabinoids. Endocannabinoids are synthesized on demand from membrane lipids and rapidly inactivated by enzymatic hydrolysis. PE—phosphatidylethanolamine, PIP2—phosphatidylinositol 4,5-biphosphate, NAPE-PLD—N-acyl phosphatidylethanolamine phospholipase D, FAAH—fatty acid amide hydrolase, NAAA—N-acylethanolamine acid amidase, MAGL—monoacylglycerol lipase, ABHD6/12—α/β-hydrolase domain-containing 6/12.

Endocannabinoids are synthesized by neurons and are degraded by both neurons as well as astrocytes [23,24]. Exogenous cannabinoids and endocannabinoids bind to the same cannabinoid receptors. From the discovery of endocannabinoids in the 1990s to the present, advances in our understanding of the endocannabinoid system have been made by identifying endocannabinoid physiology (Figure 1). NAE, or N-acylethanolamine, is a class of fatty acid amides that includes endocannabinoids like anandamide (AEA) and 2-AG. NAEs are a group of lipids characterized by a fatty acid linked to ethanolamine. N-acylphosphatidylethanolamine (NAPE) and NAE are lipid molecules involved in various biological processes, including endocannabinoid signaling and potential roles in metabolic regulation and neurodegeneration. NAPE is a phospholipid, while NAE is derived from NAPE and acts as a signaling molecule.

Anandamide, for example, is an endocannabinoid that binds to cannabinoid receptors (CB1 and CB2) similarly to how THC (tetrahydrocannabinol) from cannabis does. Anandamide, along with other NAEs, are formed in a two-step process (Figure 1): first, N-acylation of phosphatidylethanolamine generates NAPE via Ca^2+^-dependent N-acetyltransferase and, second, a phosphodiesterase of the phospholipase D type (NAPE-PLD) causes the release of NAE from NAPE [25]. DAGL-alpha, the enzyme which synthesizes endocannabinoids, is located in the dendritic spines of hippocampal cells and Purkinje cells of the cerebellum [6]. DAGL-alpha (Diacylglycerol lipase-alpha) plays a crucial role in the biosynthesis of 2-AG. Specifically, it hydrolyzes diacylglycerol to produce 2-AG. DAGL-alpha is primarily found in the central nervous system and is involved in various processes, including synaptic plasticity, neurogenesis, and the regulation of axonal growth. The activation of phospholipases results in the generation of endocannabinoids which can cause changes to synaptic activity in a retrograde fashion for tens of seconds, causing short- and long-term changes to synaptic transmission [6,14]. Chloride gradients play a key role in whether endocannabinoid-induced synaptic depression results in the reduction or enhancement of neural synaptic activity [26]. Other reviews on endocannabinoids have provided detailed information on cannabinoid receptors, the biochemistry of endocannabinoids, endocannabinoid short-term depression and long-term depression, subcellular distribution of endocannabinoids, and the physiological roles of endocannabinoids and that information will not be repeated here [6]. A greater understanding of the role that the endocannabinoid system plays in key brain regions such as the olfactory system provides new routes for the treatment for brain disorders including substance addiction [15].

The endocannabinoid hydrolyzing enzyme fatty acid amide hydrolase (FAAH) is a serine hydrolase enzyme that plays a crucial role in the breakdown of endocannabinoids and other fatty acid amides (Figure 1). It catalyzes the hydrolysis of anandamide into arachidonic acid and ethanolamine, effectively inverting its activity. FAAH is also involved in the degradation of other lipid mediators like oleamide and N-acyltaurines. In the hippocampus FAAH has been shown to be present in the somata and dendrites of cells postsynaptic to cells which have CB1R. This helps to clarify the retrograde signaling mechanism of the endocannabinoid course of action [27]. An inhibitor of FAAH has been shown to increase endogenous endocannabinoids such as anandamide. When five (5) types of FAAH were studied to determine their effect on a learning and memory task, non-matching to a procedure task in rats, one of five FAAH inhibitors, AM3506, resulted in a decrease in accuracy, whereas the other four, URB597, URB694, PF-04457845, and ARN14633, had no effect, pointing to the need for further study of how FAAH inhibitors act and the role of a more activated endocannabinoid system on learning and memory tasks [28]. The endocannabinoid system modulates immune system cells including mesenchymal such as fibroblasts which are related to cartilage erosion that occurs during rheumatoid arthritis. The presence of endocannabinoids including anandamide was shown to reduce inflammatory cytokines in human synovial fluid tissue as well as reducing rheumatoid arthritis-related arthritis in mice [29]. Glucagon-producing alpha cells of the pancreas are activated by endocannabinoid 2-AG which results in the recruitment of insulin-producing beta cells via CB1R activation in mouse fetuses and human pancreatic islet tissue, highlighting that endocannabinoids are involved in pancreatic islet cell proliferation and glucose utilization [30].

## 3. Structural and Functional Organization of the Olfactory Bulb

**Figure 2:**
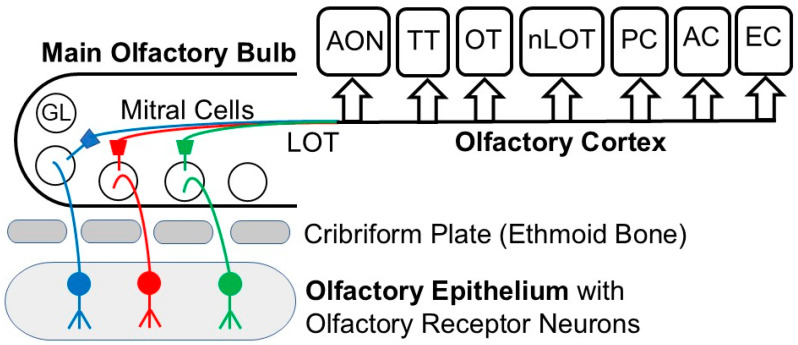
Diagrams of the olfactory pathway. GL—glomerulus, LOT—lateral olfactory tract, AON—anterior olfactory nucleus, OT—olfactory tubercle, PC—piriform cortex, EC—entorhinal cortex, AC—amygdaloid complex, TT—tenia tecta, nLOT—nucleus of lateral olfactory tract. Modified from [20].

Olfactory sensory neurons project their axon to the ipsilateral olfactory bulb where the axon terminals can form synapses with several cell types in olfactory glomeruli such as mitral cells, external tufted cells (ET), periglomerular cells and short-axon cells (SA cells) which are long-range connecting cells. Output neurons from the olfactory bulb such as mitral and tufted cells project to distinct targets in higher order centers of the brain (Figure 2). Certain SA cells are dopaminergic (DA) and GABAergic. In one group of dopaminergic, GABAergic SA cells, each cell projects to five to twelve glomeruli, and hence they are called “oligoglomerular”. Of these, one third receives direct input from the olfactory receptor nerve, ON → SA, whereas the other two thirds receive indirect input from the olfactory receptor nerve, ON → ET → SA. A second set of dopaminergic, GABAergic SA cells sends their projections to tens to hundreds of glomeruli in an extensive network; hence, these are called “polyglomerular” [31]. Dendrodendritic microcircuits within olfactory bulb glomeruli, specifically those of periglomerular cells, upon releasing GABA, are involved in the inhibition of principal tufted cells, retrograde inhibition of sensory input and lateral signaling onto neighboring principal cells [32]. There are at least two functionally distinct GABAergic circuits in the olfactory bulb that contribute to olfactory coding which include both a phasic and tonic pulse [33].

External tufted cells have extensive dendrites which project to one or sometimes two glomeruli. External tufted cells have low threshold calcium-dependent firing as well as sodium channel generated firing in response to olfactory nerve stimulation. ET cells exhibit intrinsically generated rhythmic bursts of action potential firing (~1–8 action potentials/s). ET firing becomes entrained when the olfactory nerve is stimulated [34]. ET cells that innervate the same glomerulus exhibit synchronized firing. This is achieved by coordinated synchronized synaptic transmission and gap junction coupling [35]. Even though ET cells are spontaneously active, their activity is controlled by both excitatory and inhibitory inputs to ET cells [36]. Like mitral cells, ET cells receive monosynaptic input from the olfactory nerve terminals. ET spontaneous burst firing persists even when synaptic transmission is blocked and is controlled by Na^+^, Ca^2+^, and K^+^ currents culminating in a burst of action potentials [37]. At least two functionally distinct GABAergic circuits exist in the olfactory bulb, which contribute to olfactory coding and include both a phasic and tonic pulse [33].

## 4. The Endocannabinoid System, Olfaction, and Behavior

The endocannabinoid system is expressed throughout the brain with key functions and structures being memory, reward and addiction, motor coordination, pain perception, feeding and appetite, coping with stress, schizophrenia and epilepsy. Due to the broad distribution of the endocannabinoid system throughout the brain, both the therapeutic as well as possible adverse effects and addictive potential of pharmaceutical-based endocannabinoid treatments should be studied [38]. Cannabinoid receptors are highly expressed in the main olfactory bulb and have a direct effect on olfactory bulb synaptic circuitry [18,19,20].

The appetite-enhancing effect of *Cannabis sativa* has been known from ancient times. Recent studies have confirmed the involvement of the endocannabinoid system in maintaining energy balance through the involvement of (1) the limbic system (the hedonic evaluation of foods), (2) hypothalamus and hindbrain (integration systems), (3) intestinal tissue, and (4) adipose tissue reinforcing the interaction between the endocannabinoid system and energy balance. Furthermore, the endocannabinoid system plays a role in oral motor control of suckling in newborns due to the presence of endocannabinoids in maternal milk and the activation of the CB1R [39]. The internal state of an organism is an important modulator of perception and behavior. The link between hunger, olfaction and feeding behavior is one of the clearest examples of these connections [16,40]. The endocannabinoid system has been shown to link these three behaviors: (1) hunger (need) with (2) olfaction (sense) and (3) food intake (behavior) [16]. The endocannabinoid system has been shown to be important for the behavior of olfactory foraging and novel exploration tasks, which are both compromised upon the dysfunction of the endocannabinoid system [41]. Quantitative autoradiography revealed that the binding of a CB1 receptor ligand was elevated following three weeks of HF (high fat feeding) in areas including the medial/ventral anterior olfactory nucleus, agranular insular cortex, and the hypothalamus compared to LF (low fat) controls [42]. Endocannabinoid activity is beneficial when access to food is scarce or unpredictable. However, when food is plentiful, the endocannabinoid system favors obesity and metabolic disease [43]. The endocannabinoid system, i.e., 2-AG levels, increase in sustentacular cells, the support cells in the olfactory epithelium, when animals are hungry, which points to an involvement of the endocannabinoid system in boosting the sensory inputs of food when an animal is in a state of hunger [44]. Agonists of CB1R are thought to increase appetite while inverse agonists decrease appetite. Some aromatic compounds stimulate CB1R and are thought to be a route through which food intake can be controlled more effectively [45].

The endocannabinoid system is involved in supporting the emotional and motivational factors that food stimuli elicit which contribute to psychological factors, hunger, and cravings leading to eating and overeating beyond simply eating to sustain energy needs [46]. Hunger leads to increased sensory perceptions leading to the behavior of food intake. The body’s energy needs to activate the brain which signals the main olfactory bulb to become activated which results in an increase in odor sensitivity via the activation of the CB1 receptor in the main olfactory bulb [16]. Endocannabinoids are synthesized in olfactory receptor neurons and sustentacular cells of the olfactory epithelium. 2-AG production in sustentacular cells has been shown to depend on the animal’s hunger state. The effect of 2-AG on the olfactory receptor neurons is to determine the odor receptor threshold via the CB1 receptor [44]. The data indicate that the endocannabinoid system, and particularly CB1 receptor signaling, appears to be highly significant for the mediation of hedonic aspects of reward processing [47,48]. Advanced cancer patients treated with THC reported better chemosensory perception and that food tasted better, as well as experiencing an increased caloric intake and a better quality of sleep [49]. Eating provides a strong stimulus for food consumption resulting in psychological drive. Obese subjects were found to have a lower olfactory capacity compared to non-obese ones. Furthermore, elevated fasting plasma 2-AG levels correlate with lower olfactory capacity [50]. The link between obesity and body mass was shown such that human subjects with a higher body mass index have a lower olfactory threshold discrimination identification (TDI) and higher levels of fasting plasma 2-AG levels [50].

Endocannabinoid levels have been shown to be altered in the olfactory bulb during mating in rats [51]. Studying the relationship between the olfactory bulb and endocannabinoids has shown to be a potential target for understanding and treating psychological disorders. Drugs that enhance 2-AG signaling, such as 2-AG degradation inhibitors, might be useful in human brain disorders modeled by bilateral olfactory bulbectomy such as depression and schizophrenia [52]. Social interaction impairment (SII) behavior in rats was studied with intra piriform injection of the dopamine receptor family D1R/D5R agonist SKF38393, the D2R/D3R/D4R agonist quinpirole, or both. This was done with or without pretreatment with dopamine receptor antagonists, D1R or D5R antisense oligonucleotides, the cannabinoid CB1 receptor antagonist AM281, or the endocannabinoid transporter inhibitor VDM11. The results indicate that social interaction impairment induced by the coactivation of PirC D1R and D2R requires the endocannabinoid system [53]. In another study in rats, THC and the fatty acid amide hydrolysis inhibitor URB-597 were examined in auditory and olfactory go/no-go discrimination tasks. The authors found impairment of cognitive flexibility, specifically reversal learning, by cannabinoids. They observed noteworthy sensitivity of auditory discrimination performance to THC and enhanced endocannabinoid signaling produced by URB-597 [54].

During a study of hedonic eating in healthy volunteers in which volunteers ate beyond satiation, it was found that levels of the endocannabinoid 2-AG along with ghrelin were increased, while interestingly there was a decrease in levels of the endocannabinoid anandamide as well as anandamide-related mediators oleoylethanolamide and palmitoylethanolamide when both pleasurable and non-pleasurable isoenergetic foods were eaten, showing the involvement of the endocannabinoid system in hedonic eating, i.e., eating for pleasure behavior [48]. Because endocannabinoids are an important stress buffer and are important for emotion and cognition function, the endocannabinoid system is being studied for the relationship between the malfunction of the endocannabinoid system and psychiatric disorders such as attention deficit disorder, anorexia nervosa, bulimia nervosa, and post-traumatic stress disorder, specifically through the understanding of the polymorphisms of CB1R and FAAH [55].

## 5. The Endocannabinoid System and Neurodevelopment in the Olfactory System

Neurogenesis in the olfactory system begins early in development and continues throughout life, making it a unique model for studying neuronal production, migration, and circuit integration. During embryogenesis, neural progenitors originating in the ventricular zone give rise to olfactory bulb interneurons that migrate radially and tangentially to populate emerging bulb layers, guided by a coordinated interplay of transcription factors, morphogens, and extracellular cues [56,57,58,59,60]. After birth, this process becomes spatially restricted to the subventricular zone (SVZ), where neural stem cells generate neuroblasts that migrate tangentially along the rostral migratory stream (RMS) to the olfactory bulb. Key guidance signals regulating this migration include Slit–Robo repulsive signaling, polysialylated NCAM-mediated cell–cell interactions, integrins and extracellular matrix molecules, and chemotropic cues such as brain-derived neurotrophic factor (BDNF), fibroblast growth factor (FGF), and semaphorins [61,62,63,64]. Upon arrival in the olfactory bulb, neuroblasts detach from chain migration, switch to radial migration, and differentiate primarily into granule and periglomerular interneurons, where they form functional synapses and contribute to odor processing and plasticity [65,66,67].

In adult rodents, olfactory bulb neurogenesis proceeds through distinct phases: proliferation in the SVZ, long-range migration along the RMS, activity-dependent survival, and synaptic integration, each governed by molecular mechanisms that differ from those operating during development and may even exert opposing effects [68]. Adult-born neurons are particularly sensitive to sensory experience and behavioral context, with odor enrichment promoting survival and deprivation leading to selective apoptosis [69,70]. Importantly, immature SVZ-derived neuroblasts retain the capacity to deviate from the RMS and migrate toward sites of brain injury, including ischemic lesions and traumatic damage, highlighting their relevance for endogenous repair mechanisms [71,72,73]. Guidance cues implicated in this redirection include inflammatory cytokines, chemokines such as CXCL12/CXCR4, growth factors, and extracellular matrix remodeling signals. Within this framework, the endocannabinoid system has emerged as a modulatory regulator across multiple neurogenic stages. CB1 and CB2 receptors, along with endocannabinoid-synthesizing and -degrading enzymes, are expressed in SVZ progenitors and migrating neuroblasts, where endocannabinoid signaling promotes proliferation, enhances motility, and regulates nucleokinesis and leading-process dynamics [16,74,75]. The inhibition of diacylglycerol lipase or blockade of cannabinoid receptors reduces neuroblast migration, whereas enhanced 2-AG signaling facilitates migration and integration into the olfactory bulb, effects that are particularly prominent in adulthood and aging [16,76]. These findings position endocannabinoids as context-dependent regulators of olfactory neurogenesis, with distinct roles during development, adulthood, and injury, and underscore their potential relevance for harnessing endogenous repair pathways in the damaged brain.

Following birth, neural stem cells in the SVZ continue to generate neuroblasts that travel through the rostral migratory stream and ultimately differentiate into interneurons within the olfactory bulb. This migration is essential for ensuring that newly produced neurons are incorporated correctly into established neural circuits. Throughout development and into early postnatal life, neural stem cells located in the SVZ give rise to neuroblasts that travel along the rostral migratory stream before reaching the olfactory bulb. Many molecules are known to influence this migratory journey. Recent studies have focused on their specific roles in guiding cells through the intact rostral migratory stream. Zhou et al. conducted an analysis of how endocannabinoid signaling, brain-derived neurotrophic factor (BDNF), and fibroblast growth factor receptor (FGFR) affect neuroblast motility and directional control [75]. The authors examined whether their actions differ across distinct regions of the rostral migratory stream. They found that blocking cannabinoid receptors markedly reduced cell motility and disrupted directional movement. Similar deficits occurred when the synthesis of endocannabinoids was inhibited with diacylglycerol lipase (DAGL) blockers, indicating that endocannabinoid signaling is essential for guided migration at both the entry and exit points of the rostral migratory stream. The inhibition of BDNF signaling produced comparable impairment in movement and orientation throughout the entire pathway. In contrast, manipulating FGFR activity diminished motility and altered guidance only at the proximal portion of the rostral migratory stream. In vivo FGFR blockade also led to a graded shortening of the leading processes of migrating neuroblasts along the rostral migratory stream. These findings demonstrate that all three signaling systems contribute to neuroblast navigation in the intact rostral migratory stream, with FGFR signaling displaying a distinctive region-specific requirement [75].

The endocannabinoid system increases newborn neurons in the olfactory bulb of young adult animals, while also stimulating newborn neurons in older animals [76]. More specifically, ependymal and proliferating cells in the adult mouse subventricular zone, the site of neurogenesis, express enzymes that synthesize cannabinoid receptor ligands such as DAGLs. When these enzymes and CB2 receptors are antagonized, the proliferation of cultured neural stem cells and progenitor cells in young animals is inhibited. In contrast, proliferation is enhanced when CB2 receptor agonists are applied in vivo, especially in younger animals [76]. The enzyme fatty acid amide hydrolase (FAAH) breaks down cannabinoids such as anandamide. When FAAH is inhibited and the breakdown of cannabinoids is prevented, proliferation is increased, and more neuroblasts migrate from the subventricular zone to the olfactory bulb [76]. The application of a CB2 receptor antagonist results in opposite effects with fewer newborn neurons reaching the olfactory bulb, whereas CB2 receptor agonists increase the number of newborn neurons in older animals. These observations are relevant in terms of naturally occurring reduced adult neurogenesis during aging processes [76]. Other results from the same lab confirmed the role of the endocannabinoid system in neurodevelopment [77]. Neural stem cells in the adult brain divide within the subventricular zone and give rise to neuroblasts that normally migrate along the rostral migratory stream to supply the olfactory bulb with newly generated neurons. Because these immature cells can also move toward sites of brain damage, identifying the signals that guide their movement is relevant for potential repair mechanisms. This study [77] focused on the involvement of the endocannabinoid system in the migratory phase of neuroblasts. Neuroblasts in the mouse rostral migratory stream express cannabinoid receptors as well as the enzymes DAGL-α, which produces the endocannabinoid 2-AG, and monoacylglycerol lipase, which degrades it. Using a wound-healing assay in a neural stem cell line and explant preparations from the rostral migratory stream, the authors observed that blocking either DAGL activity or CB1/CB2 receptors markedly reduces neuroblast motility. Conversely, stimulating cannabinoid receptors or limiting 2-AG breakdown enhances their migration. Time-lapse imaging of primary neuroblasts shows that the eCB system dynamically regulates nucleokinesis and shapes the length and branching patterns of migratory processes. Comparable effects occur in vivo, where GFP-labeled neuroblasts in brain slices from mice treated with CB1 or CB2 antagonists display altered morphology. Together, these results identify a previously unrecognized function of endocannabinoid signaling in directing neuroblast migration in the adult brain, underscoring its broader significance in the regulation of adult neurogenesis [77].

The endocannabinoid system is also important for developmental processes outside of the olfactory bulb and in non-rodent species, such as the development of olfactory placodes during the embryonic and larval stages of *Xenopus laevis* and for olfactory sensory neuron and basal cell development [78,79]. CB1R has been found within the olfactory epithelium of canine embryos [80]. The olfactory epithelium continues to produce new neurons throughout life and the mechanisms that govern this ongoing neurogenesis remain under continued study. Hutch and Hegg investigated how cannabinoid signaling influences the generation of new cells in the mouse olfactory epithelium [81]. Using both C57BL/6 mice and strains lacking cannabinoid receptors CB1 and CB2, the authors evaluated proliferation and lineage outcomes after administering cannabinoids either directly (WIN 55,212-2 or 2-arachidonylglycerol ether) or indirectly by blocking enzymes responsible for endocannabinoid degradation. In neonatal and adult animals, cannabinoid exposure led to an increase in proliferating cells, whereas this effect was absent in adult mice deficient in both cannabinoid receptors. Furthermore, pretreatment of adult mice with the CB1 antagonist AM251 reduced the proliferative response to cannabinoids. Although cannabinoids stimulated cell division, the number of newly formed neurons or non-neuronal cells was unchanged 16 days later. Cannabinoid treatment instead produced a transient rise in apoptosis at 72 h after treatment, with levels returning to baseline by day 16. Their observations suggest that cannabinoids enhance proliferation without promoting the formation of mature neuronal or non-neuronal cell populations, and that cannabinoid receptor signaling may influence the equilibrium between progenitor cell survival and division within the adult olfactory epithelium [81,82]. A similar pattern occurs in neural stem cells exposed to eicosapentaenoic acid, which elevates 2-AG concentrations and triggers CB1/CB2 receptor activity along with p38 MAPK (mitogen-activated protein kinase) signaling. The p38 MAPK pathway is a signaling cascade that responds to stress, such as UV light, oxidative stress, and inflammatory cytokines, and regulates cellular processes like inflammation, apoptosis, and cell cycle progression. Consistent with this, progenitor cells derived from CB1 knockout mice exhibit reduced self-renewal and proliferative capacity (reviewed in [82,83]).

The migration of neuroblasts from the SVZ to the olfactory bulb is essential for ensuring that newly produced neurons are incorporated correctly into established neural circuits. Numerous extracellular cues that influence neuroblast movement have been identified, while the intracellular pathways that govern this process remain less clear. Work by Sonego et al. [84] demonstrates that the actin-bundling protein fascin is strongly upregulated in migrating neuroblasts derived from the mouse SVZ. Mice lacking fascin-1 show marked abnormalities in the organization of the rostral migratory stream and exhibit a reduced olfactory bulb size. Bromodeoxyuridine incorporation studies, to quantify newly born cells in the brain, indicate that fascin deficiency diminishes neuroblast migration without noticeably altering cell proliferation. Loss of fascin also disrupts the polarized morphology characteristic of migrating rat neuroblasts. Their findings identify fascin as a key determinant of neuroblast movement and suggest that a finely tuned cycle of phosphorylation and dephosphorylation, shaped by external cues, is required to maintain neuroblast polarity and support effective neurogenesis [84].

While some progress has been made, the mechanisms that govern neurogenesis after brain development are still being clarified [85]. This process includes the division of neural stem and progenitor populations, their migration to appropriate regions, and their eventual differentiation and incorporation into existing neural circuits. A range of internal signaling pathways and external factors, such as neurotrophins, neurotransmitters, cytokines, and hormones, shape how these cells proliferate and mature. Endocannabinoids and their synthetic analogs have emerged as important regulators of these neural events. Growing evidence shows that the activation of CB1 and CB2 receptors influences multiple stages of neonatal and adult neurogenesis. Over the past decade, extensive research has begun mapping the cellular and molecular pathways through which cannabinoids impact neural development. The endocannabinoid system affects the differentiation and maturation of neural progenitor cells through both intrinsic and extrinsic signaling mechanisms targeted by cannabinoids. The cannabinoid system is thereby a key contributor to neurogenesis and offers new insights into neurogenic processes that persist in the mature mammalian brain [82,83,84,85].

## 6. The Endocannabinoid System and Olfactory Bulb Physiology

The link between hormone distribution and olfactory perception is providing insight into how to combat metabolic diseases that are a result of a lack of homeostasis. DAGL-alpha, the main biosynthesizing enzyme of endocannabinoids, exists in the olfactory bulb as well as other forebrain structures [76,86]. The immunochemistry of the rat brain shows that large principal cells, such as mitral cells in the olfactory bulb, show some of the strongest expressions of FAAH, the enzyme that catalyzes endocannabinoids present in the rat brain [87,88]. The presence of N-acyltransferase, an enzyme involved in the biosynthesis of N-arachidonoyl phosphatidylethanolamines (NArPEs or N-arachidonoyl PE) which is a precursor for anandamide, is present in the rat olfactory bulb [89]. NArPEs are a group of glycerophospholipids that serve as the biological precursors to anandamide. Acute stress is able to increase endocannabinoid system activity in the olfactory bulb, resulting in inhibiting GABA release in the olfactory bulb [90]. An N-acyl phosphatidylethanolamine phospholipase D (NAPE-PLD) that catalyzes the formation of NAEs was shown to be highly present in the axons of the vomeronasal nerve which projects to the accessory olfactory bulb, suggesting that NAEs, such as the endocannabinoid anandamide, act as an anterograde synaptic signaling molecule [27].

Cannabis extract reduces epileptiform type bursting in the rat olfactory cortex [91,92]. Cannabis extract and Delta9-THC act via CB1 receptors and inhibit muscarinic agonist-induced epileptiform bursting in rat olfactory cortical brain slices. The natural compounds, sesamol and curcumin, both similar to amitriptyline, a medication used in the treatment of depression and anxiety, increase both neurotrophins and endocannabinoid concentration in the brain [93].

**Figure 3:**
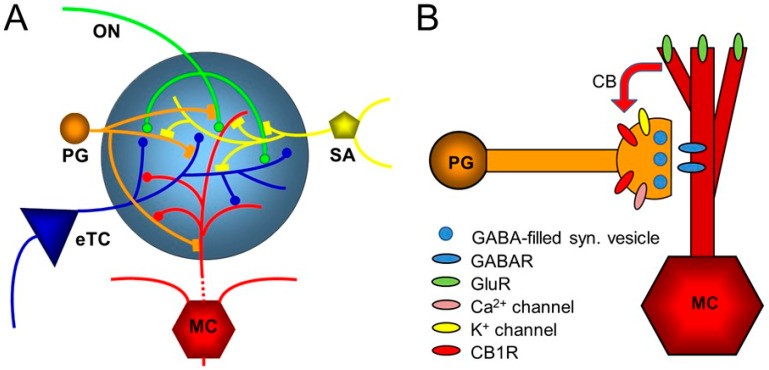
Diagram of the glomerular network. (**A**) Olfactory nerve (ON) afferents enter the main olfactory bulb through the olfactory nerve laver to synapse with periglomerular cells (PGs), mitral cells (MCs), and tufted cells (of which only external ones, eTCs, are shown) within the glomerular layer. Periglomerular cells inhibit olfactory nerve terminals, external tufted cells, and mitral cells. The processes of short-axon (SA) cells, which are GABAergic and dopaminergic, receive excitatory synaptic input and form extensive interconnections between glomeruli. Mitral cell apical dendrites convey sensory information to deeper layers of the main olfactory bulb. Mitral cells and tufted cells form dendrodenritic synapses with glomerular neuronal processes. (**B**) Dendrodendritic interactions of mitral cells and periglomerular cells. Cannabinoids are released non-synaptically by mitral and potentially other cells and act on cannabinoid receptors in periglomerular cells to modulate their synaptic release of GABA. Only the apical dendrite of the mitral cell is shown. GABAR—GABA receptor, GluR—ionotropic and metabotropic glutamate receptor. From ref. [20].

Figure 3 shows the glomerular network and synaptic connections between cells that are regulated by endocannabinoids. Table 1 lists the distribution of CB1 receptors in the main olfactory bulb and the effect of endocannabinoid modulation in the circuit.

## 7. Endocannabinoid Receptor and Vanilloid Receptor

The vanilloid receptor (TRPV1 or VR1) is a molecular integrator of various painful stimuli, including capsaicin, acid, and high temperature, and can be activated by the CB1R agonist anandamide. TRPV1 has been found to be present in the olfactory bulb [96]. The vanilloid receptor, which upon binding to its ligand capsaicin is used to relieve neuropathic pain, uremic pruritus, and bladder overactivity, has also been shown to bind to sensory neurons since vanilloid receptors are found in the olfactory bulb [97,98]. All major cell types involved in the cerebrovascular control pathway—endothelium, smooth muscle, neurons, pericytes, and microglia—have been shown to produce endocannabinoid-related proteins, including CB1 and CB2 receptors as well as transient vanilloid type 2 receptors. The endocannabinoids have been shown to play a role in the cerebrovascular system, specifically causing vasodilation via the relaxation of smooth muscle, and the release of vasodilation mediators from the endothelium. Also, the endocannabinoid system has been shown, during brain pathologies such as subarachnoid hemorrhage, trauma and ischemic brain injury, to activate CB2 receptors resulting in greater blood perfusion. However, during stress conditions (restrained conscious animals experiencing hypoxia or hypercapnia) the result was a decrease in cerebral blood flow [99].

Compounds which bind to the vanilloid receptor TRPV-1, a relative of the cannabinoid receptor, such as capsaicin, have been studied for the protective role that TRPV-1 activation plays in oxidative stress on keratinocytes and human blastoma cells in vivo, as well as the protective role it plays in cytotoxicity and analgesia [100]. FAAH/TRPV1 and FAAH/COX-2 inhibitors interact with both endocannabinoid and endovanillanoid systems. Both systems are regulated by lipid mediators, i.e., prostaglandins produced by COX enzyme, pointing to the interconnectedness of both systems [101]. Anandamide has been shown to inhibit squamous cell proliferation. PUFA, poly-unsaturated-based enthanolamines like the endocannabinoid anandamide, D-docosahexaenoyl enthanolamides (DHEA), and N-arachidonoyl-L-alanine (NALA) have both been shown to inhibit head and neck squamous cell carcinoma line proliferation (HNSCC). However, their action was thought to work in an endocannabinoid system-dependent fashion. When both endocannabinoid receptors, CB1R and VR1, were inhibited, this inhibition of cellular growth did not persist [102]. The endocannabinoid receptor TRPV1 has been reviewed as a potential anti-nausea and anti-vomiting treatment target because it has been implicated in the pathways of nausea and vomiting along with other diverse transmitter systems including acetylcholine, dopamine, endorphins, glutamate, histamine, 5-hydroxytryptamine, and substance P [103]. Based on the experience of altered senses in people with advanced cancer and the impact on their quality of life including an impaired ability to perceive and appreciate sensations, it has been suggested that additional research should be carried out to identify how senses are perceived in order to determine how to overcome the chemosensory altered experiences of cancer patients. Alterations can include persistent bad tastes, phantom smells, and hypersensitivity to odors and food flavors. For the purpose of overcoming these chemosensory alterations, more studies of sensory science should be done. The presence of cannabinoid receptors in the olfactory bulb could prove to be helpful in bridging our understanding of the endocannabinoid involvement in chemosensory processing [49].

## 8. Therapeutic Potential of Endocannabinoids

The endocannabinoid system in the olfactory pathway is unusually translatable. The translational/therapeutic angle becomes more obvious when we think about olfaction as not just smell. In mammals, it is tightly coupled to feeding, reward, stress state, and exploration, and the endocannabinoid system is one of the systems that links internal state to circuit gain. A clear example was shown by CB1 signaling in centrifugal inputs to the main olfactory bulb (MOB) which increases odor detection in fasted mice and promotes feeding, providing a mechanistic bridge from circuit modulation [16,20].

More broadly, cannabinoid modulation of olfactory processing is now documented across multiple olfactory structures (main olfactory bulb and cortex) and behavioral readouts [94,104]. One explicit therapeutic opportunity is in appetite and metabolic disorders (state-dependent olfactory “gain control”). If the endocannabinoid tone in olfactory circuits helps to tune odor sensitivity based on the hunger state, then targeted manipulation could, in principle, modulate maladaptive appetite cues. A potential application relates to cachexia/anorexia (enhance cue salience), obesity (reduce cue-driven intake), and conditions where appetite is distorted by sensory processing. The link between main olfactory bulbs, CB1 receptors, and feeding supports this idea mechanistically [16,40]. The caveat with this translational aspect is that systemic CB1 blockade “worked” for weight loss but had unacceptable psychiatric side effects in real-world development (rimonabant’s history is the classic example) [105,106]. Rimonabant was the first selective CB1 receptor blocker developed for treating obesity and related cardiometabolic risk factors, such as type 2 diabetes and dyslipidemia. Clinical trials showed that it was effective in producing modest but sustained weight loss and improving metabolic profiles. It also showed potential for smoking cessation. European regulatory authorities, specifically the European Medicines Agency (EMA), approved rimonabant (marketed as Acomplia and Zimulti) in 2006. The drug was quickly withdrawn from the European market in 2008 due to the emergence of significant and serious adverse psychiatric effects. These side effects, which included severe mood disorders, depression, anxiety, and suicidal ideation, occurred at a rate significantly higher than in placebo groups. This drug failure motivates circuit- or region-biased strategies rather than whole-brain CB1 antagonism.

Olfactory dysfunction and circuit repair (neurogenesis and plasticity) is another translational aspect with respect to endocannabinoids. The olfactory bulb is one of the canonical targets of adult-born interneurons from the subventricular zone [107]. The endocannabinoid system has been implicated in neuroblast migration and adult neurogenesis steps, which makes it reasonable to contemplate “repair” hypotheses for some forms of smell dysfunction [77,108]. These findings suggest a plausible mechanistic lever for recovery-oriented interventions, but whether this can be harnessed safely and effectively in humans remains an open question.

Another translational aspect relates to neuroinflammation and neurodegeneration where smell is an early symptom, even though, here, the emphasis is on CB2 receptors. Olfactory regions are early-affected nodes in several neurodegenerative trajectories, and endocannabinoid system components (especially CB2 receptors on glia) are attractive anti-inflammatory targets. Several studies have highlighted CB2 as potentially neuroprotective and a candidate biomarker/target [109,110,111]. CB2-focused approaches are often discussed as having less psychoactive liability than CB1-heavy strategies, since CB2 expression is enriched in immune/glial contexts [110,112].

Access to the olfactory system through direct nose to brain delivery is a practical therapeutic angle. Because the olfactory pathway is literally the access route, intranasal delivery is a translational strategy to bias exposure toward olfactory/trigeminal routes and potentially reduce systemic exposure. The rationale is that intranasal administration can reach the brain via olfactory and trigeminal pathways and is heavily studied as a CNS delivery route [113,114,115]. Cannabinoid-relevant examples include the intranasal CBD formulations that have been engineered to improve brain targeting in animal models (e.g., hydrogels, nanoparticles) [116,117]. However, ADME pharmacokinetic and formulation questions remain open, such as how much actually reaches the olfactory region or brain after intranasal delivery, how fast it works, for how long and how variable it is between individuals. Hence, this strategy is promising and under active development and not a proven clinical route for cannabinoids. Safety concerns have the highest priority and are the reason why this has not already become a drug. Another translation barrier is the fact that global enzyme inhibition is not automatically safe. The catastrophic FAAH inhibitor trial (BIA 10-2474) is a cautionary tale about off-target effects and class assumptions [118,119]. The BIA 10-2474 FAAH inhibitor trial was a catastrophic Phase 1 study in France (2016) where one volunteer died, and four others suffered severe brain damage. This was caused by unexpected off-target effects on other enzymes (lipases) and altered lipid metabolism, not just FAAH inhibition, leading to significant regulatory scrutiny and halting similar drug development. Researchers found that the drug disrupted lipid networks in neurons, unlike other selective FAAH inhibitors, which highlighted the risks in first-in-human trials and the importance of broad screening. CNS side effects limit some endocannabinoid drugs, which is the reason for pursuing peripherally restricted or biased approaches, e.g., peripherally restricted MAGL inhibitors, to avoid central cannabimimetic effects [120,121].

In summary, mechanistically, the endocannabinoid system is well positioned to couple internal state to olfactory circuit gain. In the main olfactory bulb, CB1 receptor-mediated signaling in centrifugal inputs can increase odor detection and promote feeding, suggesting a pathway by which endocannabinoid system modulation could influence cue-driven appetite [16]. Beyond the main olfactory bulb, endocannabinoids shape cortical network dynamics relevant to odor detection [104]. Translationally, these findings motivate targeted strategies (region-, cell type-, or delivery-biased) for disorders where olfaction and motivated behavior are disrupted, while acknowledging that systemic CB1 manipulation has shown unacceptable psychiatric liability and that broad enzyme inhibition can carry serious safety risks [117,118]. Intranasal approaches may offer a practical route to bias exposure toward olfactory/trigeminal pathways, though clinical efficacy for cannabinoid-based nose-to-brain therapeutics remains to be established [113,116].

## 9. Methodological Limitations

Several methodological limitations can impair studies of the endocannabinoid system in the olfactory pathway, i.e., from olfactory epithelium to the main olfactory bulb, to the piriform cortex and beyond, while measures have been taken to reduce the risk of misinterpreting results [122]. Measuring endocannabinoids is difficult as they change fast and can do so during handling. 2-AG is chemically unstable and can isomerize to 1-AG during extraction/processing (pH, solvents, time), which can create misleading levels [123,124,125]. Enzymes keep working after sampling, unless they are quenched rapidly, so the “measured concentration” can reflect post-collection degradation rather than in vivo tone [123,125]. The best practice is rapid quenching, cold chain, enzyme inhibitors where appropriate, stable isotope internal standards, and reporting 2-AG and 1-AG together or explicitly prevent/track isomerization [123,124,125].

Localizing CB1 and especially CB2 with antibodies is difficult without strict validation. Many commercial CB1 antibodies have specificity problems; staining can persist even when the receptor should not be present [126,127,128]. CB2 in brain tissue is even more contentious methodologically if localized with immunohistochemistry [127,129]. Best practices include knockout/knockdown controls, multiple antibodies against different epitopes, orthogonal confirmation, e.g., mRNA methods, and being conservative in claims about cell type expression.

Pharmacological experiments can obscure olfactory experiments. In olfactory studies, cannabinoids/endocannabinoid modulation can affect arousal, anxiety, appetite, locomotion, and motivation which can be misinterpreted as improved odor sensitivity or reduced discrimination, depending on the task design, adding another level of complexity to studies of olfactory behavior [94,104,130,131,132]. Best practices include pairing odor tasks with controls for locomotion/sniffing/exploration and using task designs that separate sensory detection from decision/motivation, e.g., well-controlled go/no-go.

Enzyme inhibitors are not clean switches for AEA versus 2-AG. FAAH inhibition, e.g., with URB597, can elevate other FAAH substrates that act on channels like TRPV1/TRPV4, and can produce effects not strictly mediated by CB receptors [133]. MAGL inhibition, e.g., with JZL184, can show cross-reactivity, especially at higher doses, and chronic global MAGL inhibition can induce CB1-like dependence/withdrawal phenotypes, complicating long experiments [120,134]. Degradation of 2-AG is not only mediated by MAGL. ABHD6 and ABHD12 (alpha/beta-hydrolase domain containing 6 and alpha/beta-hydrolase domain containing 12, respectively) are enzymes in the alpha/beta-hydrolase family. They are crucial for breaking down lipids, particularly 2-AG, alongside MAGL, and play key roles in the nervous system and metabolism. They have distinct subcellular locations (ABHD6 cytoplasmic, ABHD12 extracellular/lumenal) and substrate preferences. Therefore, interpreting a manipulation such as 2-AG going up everywhere can be wrong [135,136]. It is best to triangulate with genetics (cell type specific knockouts), dose–response, short versus chronic comparisons, and measuring lipids directly when possible.

Slice physiology can miss key olfactory pathway features. Many studies of the endocannabinoid system in olfaction rely on acute brain slices. Long-range centrifugal feedback and natural sniff-driven input patterns are altered by slicing as the input and higher order olfactory centers are cut off. Endocannabinoid signaling is highly local and state-dependent, so physiological observations in the slice may not match intact neural network dynamics [18,20,130,137]. Ideally, results obtained in slices are combined with in vivo physiology/behavior when making pathway-level claims.

A recent study suggests that AEA and 2-AG have different roles by acting either on astrocytes or neurons which adds a level of complexity in understanding the role of the endocannabinoid system [95] and may confound previously obtained results attributed to neurons alone. The authors show that in the hippocampus 2-AG selectively signals to neurons, inducing depression, whereas AEA signals to astrocytes, inducing lateral synaptic potentiation. These results reveal distinct cell type-specific signaling pathways that involve unique eCBs selectively signaling to either neurons or astrocytes [95].

Methodological advances and novel tools help, but they introduce new interpretation limits. Genetically encoded sensors like GRABeCB2.0 can report fast, spatially resolved endocannabinoid dynamics, which is a significant advancement for circuit analysis in the main olfactory bulb [138,139]. However, careful pharmacological characterization matters, because sensor responses can be influenced by which ligands are present and how they interact with the sensor design [140,141].

## 10. Future Directions and Unanswered Questions

This review examines the endocannabinoid system as a key modulator of olfactory circuitry, linking internal state to sensory processing, behavior, and plasticity. It outlines the core biology of the endocannabinoid system and the organization of the olfactory bulb, highlighting how CB1-mediated signaling shapes synaptic gain, odor sensitivity, and olfactory-guided behaviors related to hunger, reward, and stress. It also reviews the evidence that endocannabinoids regulate olfactory neurodevelopment and adult neurogenesis, influencing neural stem cell proliferation, migration, and circuit integration across the lifespan. Interactions between cannabinoid and vanilloid receptors, as well as roles in neuroinflammation and disease, are discussed alongside translational opportunities and risks, including appetite modulation and intranasal delivery strategies. Methodological challenges in measuring and manipulating endocannabinoid signaling are emphasized, underscoring the need for circuit-specific and in vivo approaches.

Several questions regarding endocannabinoid function in the olfactory pathway remain unanswered and are outlined below. Where and when are endocannabinoids released during real odor sampling? Most mechanistic work is done in reduced preparations such that we still lack a spatiotemporal “map” of endocannabinoid system signals across glomeruli and deeper layers of the main olfactory bulb during sniffing and behavior. New genetically encoded sensors, e.g., GRABeCB2.0, make this question tractable in vivo [142,143,144]. Which endocannabinoid ligand is acting in olfaction: 2-AG, AEA, or both? Many studies infer the engagement of the endocannabinoid system from receptor pharmacology, but do not directly disambiguate ligand identity or compartment. Pairing lipid measurements with causal manipulations (DAGLα/β vs. NAPE-PLD/FAAH) and sensor readouts can help in this regard [142,145]. How does the modulation of the endocannabinoid system interact with corticofugal feedback to the bulb? Cortical feedback strongly shapes bulb output. The endocannabinoid system sits right in that loop (including feedback-driven excitation patterns). A key open question is whether endocannabinoids gate feedback globally (state setting) or selectively (odor- and context-specific tuning) [20,137]. How does the metabolic state recruit the endocannabinoid system in olfactory circuits? A canonical result is that CB1 signaling in centrifugal inputs can increase odor detection and promote feeding in fasted animals. What remains open is the upstream “state sensor” logic and how general this is across motivational states, diets, and species [16,19]. What is the role of CB2 (if any) in olfactory processing versus neuroinflammation? CB2 is often discussed in glial and inflammatory contexts, but brain expression patterns and functional contributions are still actively debated and can be state-dependent (e.g., inflammation). Testing CB2’s roles in olfaction likely requires cell type-specific genetic tools rather than antibody-only localization [110,146,147]. Does the endocannabinoid system regulate adult-born neuron integration into olfactory bulb circuits in a functionally meaningful way? Strong evidence exists that components of the endocannabinoid system modulate adult neurogenesis broadly, including studies of the subventricular zone, but linking that to olfactory learning, discrimination, and bulb circuit stability is still a key translational gap [75,85,148]. How does the endocannabinoid system shape network rhythms and synchrony relevant to odor coding? Output from the main olfactory bulb depends on oscillations and synchrony, and cortical feedback is known to influence these. A clear future direction is testing whether endocannabinoid signaling is a knob for rhythm selection (gamma/beta) under different tasks and states [130,137]. How does the endocannabinoid system interact with other modulators in the olfactory pathway? Olfactory circuits are heavily co-modulated (acetylcholine, noradrenaline, dopamine, nitric oxide, peptides). A concrete unanswered question is whether the endocannabinoid system integrates with these systems additively, hierarchically, or via nonlinear “gating” interactions [130,149]. Can we separate “sensory” effects from the changes in motivation/arousal in behavioral assays? Cannabinoid manipulations can shift exploration, anxiety, and motivation, which can look like sensory changes. Future work should standardize behavioral pipelines that isolate detection/discrimination from motivational confounds [16,19]. These questions open the door for exciting future studies to fully comprehend the role of the endocannabinoid system along the olfactory pathway.

## 11. Conclusions

The body of work reviewed here demonstrates that the endocannabinoid system is a fundamental regulator of olfactory circuit function, linking metabolic, emotional, and motivational states to sensory processing. Within the main olfactory bulb, endocannabinoid signaling, largely mediated by CB1 receptors localized to specific axonal and dendritic compartments rather than principal cell somata, modulates inhibitory and excitatory microcircuits that shape odor sensitivity, discrimination, and output to higher brain regions. This organization enables the endocannabinoid system to act as a dynamic gain control mechanism, adjusting olfactory processing according to the internal state, such as hunger or stress.

Beyond acute circuit modulation, endocannabinoids play a significant role in olfactory system development and plasticity. From embryonic placode formation to adult neurogenesis, cannabinoid signaling influences neural stem cell proliferation, neuroblast migration along the rostral migratory stream, and the incorporation of new interneurons into olfactory bulb circuits. These developmental and regenerative roles highlight the olfactory system as a unique window into the broader principles of endocannabinoid regulation of brain plasticity.

Importantly, the translational potential of these findings is substantial but must be approached with caution. Systemic manipulation of the endocannabinoid system, particularly global CB1 antagonism or broad enzyme inhibition, has been limited by psychiatric and neurological side effects. The olfactory pathway, however, offers opportunities for more targeted interventions, including region-biased pharmacology, cell type-specific strategies, and intranasal delivery approaches that may reduce systemic exposure. Such strategies could be relevant for disorders characterized by altered olfaction, appetite dysregulation, neuroinflammation, or early neurodegenerative changes.

At the same time, significant methodological challenges remain. Accurately measuring endocannabinoid dynamics, localizing receptors with confidence, and disentangling sensory effects from changes in motivation or arousal require rigorous experimental design and the integration of complementary approaches. New tools, such as genetically encoded endocannabinoid sensors and cell-specific genetic manipulations, now make it possible to address many of these limitations in vivo.

In conclusion, the endocannabinoid system is uniquely positioned to couple the internal state to olfactory perception through precise, circuit-level modulation. Continued investigation of this interaction promises not only to deepen our understanding of olfactory neuroscience but also to inform the development of safer, more targeted cannabinoid-based therapies for neurological, metabolic, and psychiatric disorders.

## Figures and Tables

**Table 1:** CB1 receptor expression in the main olfactory bulb (MOB). CB1R expression is strongest on specific axonal/process compartments (especially GL “periglomerular processes” and GCL centrifugal terminals) and not broadly on principal cell somata. The horizontal limb of the diagonal band (HDB), part of the basal forebrain, sends significant GABAergic (inhibitory) and cholinergic (excitatory/modulatory) projections to the MOB, critically modulating how the MOB processes odors by influencing mitral/tufted cells (output neurons) and interneurons, impacting sensitivity, discrimination, and tuning to specific smells, essentially balancing excitation and inhibition to sharpen olfactory perception. EPL—external plexiform layer, ECS—endocannabinoid system. Based on [15,16,17,20,94,95].

MOB Element/Cell Type	Where in MOB	CB1R Localization (Cell Body vs. Dendrites vs. Axons/Terminals)	Notes
Mitral cells	Mitral cell layer (MCL); apical dendrites in GL; lateral dendrites in EPL	Not detected in mitral cells; mitral cell output is modulated indirectly via CB1 on glomerular interneuron processes	CB1 present in periglomerular processes but not in mitral cells; effects disappear when GL is removed, supporting a glomerular locus.
Tufted cells (incl. external tufted)	Tufted somata in EPL/GL (external tufted in GL); dendrites in GL/EPL	No strong CB1 signal on tufted cell bodies is emphasized; tufted activity is affected mainly via CB1 on PG processes (and related glomerular circuitry)	Cannabinoids have strong direct effects on PG cells, weak on external tufted cells, consistent with CB1 mainly on the inhibitory side of the synapse.
Periglomerular cells (PG)	Glomerular layer (GL)	CB1 reported in periglomerular processes (a GAD65 + subset); described as process-localized rather than clearly “somatic CB1”	Multiple lines point to CB1 on GABAergic PG processes that shape inhibition onto output neurons.
Short-axon cells (SA)	Superficial SA in GL; deep SA in GCL	Best-supported CB1-related signal is at centrifugal synapses onto deep short-axon cells, consistent with CB1 on presynaptic centrifugal terminals (not necessarily on SA cell bodies)	Cortical feedback excites both granule cells and deep short-axon cells, and CB1 activation suppresses these synapses.
Granule cells (GC)	Granule cell layer (GCL); dendrites into EPL	Strongest, cleanest localization is CB1 on axon terminals of centrifugal (cortical) glutamatergic projections targeting granule cells; also, CB1 modulates other long-range inputs onto granule cells (e.g., HDB → GC)	Soria-Gómez shows CB1 abundant on centrifugal cortical glutamatergic terminals in GCL; additional work shows CB1 modulation at HDB → GC synapses.
Olfactory sensory neurons (OSNs; “sensory receptor neurons”)	Olfactory nerve layer (ONL) → glomeruli	CB1 is found primarily in glomerular interneuron processes, not as a clearly mapped receptor on OSN axon terminals	OSN terminals are strongly regulated by glomerular inhibition, and ECS effects on “sensory gain” are usually framed via the PG network/GL circuitry rather than OSN CB1 localization.
Centrifugal fibers entering the MOB (corticofugal feedback)	Predominantly synapse in GCL (also other layers depending on source region)	CB1 abundant on axon terminals of centrifugal cortical glutamatergic neurons projecting to granule cells; CB1 also modulates corticofugal synapses onto deep short-axon cells	This is one of the most explicit, repeatedly cited “where CB1 is” statements for MOB circuitry.

## Data Availability

No new data were created or analyzed in this study.
